# Psychedelic-induced hypomania and mania: a systematic review and meta-analysis

**DOI:** 10.1038/s41380-026-03657-6

**Published:** 2026-05-29

**Authors:** Mickael Eskinazi, Rayan Nasserdine, Romane M. Cusin, Pantelis Baniotoupoulos, Luigi F. Saccaro, Marco De Pieri, Tamara Corino, Federico Seragnoli, Jean-Francois Briefer, Tatiana Aboulafia Brakha, Hélène Richard-Lepouriel, Louise Penzenstadler, Kerem Böge, Matthias Kirchner, Daniele Zullino, Mikkel Højlund, Jacopo Sapienza, Marta Bosia, Ana Catalan, Eduard Vieta, Marco Solmi, Michel Sabé

**Affiliations:** 1 Division of Adult Psychiatry, Department of Psychiatry, University Hospitals of Geneva, 2, Chemin du Petit-Bel-Air, CH-1226 Thonex, Switzerland; 2 Mood Disorder Unit, Psychiatric Specialties Service, Geneva University Hospital, Geneva, Switzerland; 3 Faculty of Medicine, University of Geneva, Geneva, Switzerland; 4 Fireside Lab, Fireside Project, Venice, CA 90291 Italy; 5 Division of Addiction Psychiatry, Department of Psychiatry, University Hospitals of Geneva, 70, Grand-Pré, CH-1202 Geneva, Switzerland; 6 Department of Psychiatry and Neurosciences, Charité Universitätsmedizin Berlin, Campus Mitte, Berlin, Berlin, 10117 Germany; 7 German Center for Mental Health (DZPG), partner site Berlin/Potsdam, Potsdam, Berlin Germany; 8 Medical University Brandenburg - Theodor Fontane, Neuruppin, 16816 Brandenburg Germany; 9 Department of Psychiatry Aabenraa, Mental Health Services in the Region of Southern Denmark, Aabenraa, Denmark; 10 Clinical Pharmacology, Pharmacy, and Environmental Medicine, Department of Public Health, University of Southern Denmark, Odense, Denmark; 11 Child and Adolescent Mental Health Centre, Mental Health Services Capital Region of Denmark, Copenhagen, Denmark; 12 IRCCS San Raffaele Scientific Institute, Milan, Italy; 13 Department of Humanities and Life Sciences, University School for Advanced Studies IUSS, Pavia, Italy; 14 School of medicine, Vita-Salute San Raffaele University, Milan, Italy; 15 Department of Psychiatry, Basurto University Hospital, Bilbao, Spain; 16 Biobizkaia Health Research Institute, Barakaldo, Spain; 17 CIBERSAM. Centro Investigación Biomédica en Red de Salud Mental, Madrid, Spain; 18 University of the Basque Country, UPV/EHU, Leioa, Spain; 19 Department of Psychosis Studies, Institute of Psychiatry, Psychology & Neuroscience, King’s College London, London, UK; 20 Department of Medicine, Faculty of Medicine and Health Sciences, Institute of Neurosciences, University of Barcelona, Barcelona, Catalonia Spain; 21 Bipolar and Depressive Disorders Unit, Hospìtal Clinic de Barcelona, Barcelona, Catalonia Spain; 22 Institut d’Investigacions Biomèdiques August Pi i Sunyer (IDIBAPS), Barcelona, Catalonia Spain; 23 Centro de Investigación Biomédica en Red de Salud Mental (CIBERSAM), Instituto de Salud Carlos III, Madrid, Spain; 24 SCIENCES lab, Department of Psychiatry, Faculty of Medicine, University of Ottawa, Ontario, Canada; 25 Department of Mental Health, The Ottawa Hospital, Ottawa, Ontario Canada; 26 Ottawa Hospital Research Institute (OHRI), The Ottawa Hospital, Ottawa, Ontario Canada; 27 Department of Child and Adolescent Psychiatry, Charité Universitätsmedizin, Berlin, Germany

**Keywords:** Neuroscience, Bipolar disorder

## Abstract

Serotonergic psychedelics are increasingly investigated as treatments for affective disorders. Concerns persist regarding their potential to induce hypomania or mania, particularly in individuals with bipolar spectrum vulnerability. Whether these substances precipitate transient mood switches or contribute to persistent bipolar illness or diagnostic transition remains unclear. We conducted a systematic review of human studies examining manic or hypomanic symptoms following exposure to serotonergic psychedelics (psilocybin, LSD, mescaline, DMT/ayahuasca) or MDMA (CRD420251160656). Databases and trial registries were searched through January 26, 2026. Eligible designs included randomized and non-randomized clinical studies, registry-based cohorts, cross-sectional surveys, and longitudinal observational studies. Outcomes included dysphoria/euphoria, manic or hypomanic symptoms and transition to bipolar disorder. Risk of bias was assessed using ROBINS-I, ROB2 or NIH tools. Twenty-three studies met inclusion criteria, four contributing to meta-analysis. Rates of psychedelic-associated dysphoria/euphoria, hypomania or mania ranged from 5.8% in controlled trials of psilocybin-assisted psychotherapy for major depressive disorders to 30% in naturalistic studies of individuals with bipolar disorder. When present, manic symptoms were typically acute and self-limited. Observational studies identified higher risks among individuals with bipolar I disorder, familial vulnerability, polysubstance use, and unsupervised or illegal use. Registry-based cohorts examining diagnostic transitions showed a prevalence of subsequent transition to bipolar disorder of 4% (95% CI 2–8%; N = 7478; I² = 32.1%), with little evidence for a hallucinogen-specific signal. Overall, serotonergic psychedelics appear to pose a low but clinically meaningful relative risk of transient mood-related symptoms in susceptible individuals while remaining relatively safe in controlled clinical settings. Long-term outcomes and repeated exposure remain insufficiently studied, underscoring the need for rigorous longitudinal research.

## Highlights

- Psychedelics show low overall risk of hypomania or mania in clinical settings
- Naturalistic and unsupervised use is associated with higher hypomanic/manic risk
- No evidence supports psychedelics inducing bipolar disorder de novo
- Hypomanic/manic symptoms occur mainly in bipolar I or genetically vulnerable individuals
- Psychedelic-related hypomanic, manic symptoms are typically acute and self-limited

## Introduction

Over the past two decades a resurgence of clinical interest in serotonergic psychedelics including psilocybin, lysergic acid diethylamide (LSD), dimethyltryptamine (DMT)/ayahuasca, and mescaline as well as MDMA (3,4-methylenedioxymethamphetamine), has prompted renewed investigation into their therapeutic potential for mood, anxiety, and substance use disorders [1]. Psilocybin and LSD has shown promising antidepressant effects in treatment-resistant depression and major depressive disorder, with several randomized controlled trials (RCTs) demonstrating rapid and durable symptom relief [2]. These developments, alongside growing public and legislative support [3], have accelerated the momentum toward regulatory approval and expanded clinical access.

However, while enthusiasm for psychedelic therapies has surged, concerns about adverse psychiatric reactions—especially psychotic exacerbation [4], and manic or hypomanic switches in individuals with underlying bipolar spectrum vulnerabilities—remain under-examined and poorly quantified [5]. Mania and hypomania are potential consequences of pharmacologically induced serotonergic activation, and serotonergic antidepressants such as selective serotonin reuptake inhibitors and monoamine oxidase inhibitors are well-known precipitants of treatment-emergent affective switches in bipolar disorder [6], especially in the context of antidepressant monotherapy [7]. Given that classic psychedelics act primarily as 5-HT2A receptor agonists, and with indirect effects on dopaminergic, and glutamatergic transmission, it is plausible that they may carry similar or even greater risks of inducing manic or hypomanic symptoms or episodes, particularly in susceptible individuals. Indeed, clinical and survey-based evidence suggests that psychedelics may precipitate new or worsened manic or hypomanic symptoms in some individuals with bipolar disorder or bipolar-spectrum vulnerability, although the available evidence remains limited and heterogeneous, raising questions about unidentified risk modifiers [8, 9]. Furthermore, retrospective chart reviews and registry-based data suggest that substance-induced manic episodes may evolve into primary bipolar diagnoses in a substantial subset of patients [10]. The phenomenology of psychedelic-induced mood symptoms is complex, ranging from transient symptoms (trip-related euphoria, persistent psychedelic effects) to psychedelic-induced hypomanic/manic episodes (or mood switch, or mixed states), that could ultimately lead to a transition to a bipolar disorder (Supplementary Table 1). These symptoms and episodes must also be distinguished in patients with an actual diagnosis and those with/or without personal of family history.

Modern clinical trials of psychedelics have generally excluded individuals with bipolar-spectrum disorders, particularly bipolar I disorder, as well as those with histories of mania or psychosis or a first-degree family history of bipolar disorder, although a few recent early-phase studies have begun to include carefully selected individuals with bipolar II disorder. While this approach has contributed to reassuring safety data in unipolar depression and anxiety populations, it has created a significant gap in our understanding of the risks posed to those with bipolar spectrum vulnerabilities. Consequently, existing RCTs cannot be used to infer the safety profile of psychedelics in patients with known or latent bipolar disorders. In contrast, large-scale online surveys—while subject to self-selection and recall biases—suggest that new or increased manic symptoms may be among the more frequently reported adverse effects in real-world settings. One international survey of individuals with bipolar disorder who had used psilocybin reported that approximately 14–32% experienced new or intensified manic symptoms the week following use [10]. These findings echo concerns voiced by clinicians that psychedelic substances may unmask or trigger manic episodes in a subset of users.

Given the increasing use of psychedelics outside clinical research—often in unregulated, unsupervised, or recreational contexts [11]—better characterization of bipolar-relevant adverse outcomes, particularly treatment-emergent dysphoria/euphoria, mania, and hypomania, is needed [9]. Clinical guidance remains limited [12, 13], and decision-making around the inclusion of bipolar patients in therapeutic protocols is often based on expert opinion or indirect evidence [14]. Current consensus favors avoiding psychedelic administration in individuals with bipolar I disorder or clinical high risk for psychosis, and exercising caution in those with chronic psychosis, bipolar II disorder, or a positive family history [15, 16].

Despite this caution, a comprehensive and methodologically rigorous synthesis of literature on psychedelic-associated dysphoria/euphoria, mania and hypomania is lacking. To address this gap, we conducted a systematic review of retrospective, and prospective studies to evaluate the prevalence, temporal characteristics, and risk factors associated with psychedelic- (or MDMA)-induced manic and hypomanic symptoms. We aimed to distinguish short-term versus persistent outcomes, explore potential moderating variables such as psychiatric comorbidities or family history, and assess the strength and quality of evidence across study types. MDMA was included as a related but pharmacologically distinct compound because it is often discussed within the broader psychedelic treatment literature. By consolidating current knowledge, we seek to inform clinical risk assessment and guide future research on the safe application of psychedelic treatments in mood disorder populations.

## Methods

This systematic review adheres to the Preferred Reporting Items for Systematic Reviews and Meta-Analyses (PRISMA) 2020 guideline (CRD420251160656).

### Search strategy

We conducted a systematic search in PubMed, Embase, PsycINFO, and PsycARTICLES from database inception to January 26, 2026, without restrictions on publication date or language. A comprehensive search terms combining (bipolar OR dysphoria OR euphoria OR hypomania OR manic) with psychedelics (psilocybin, LSD, ayahuasca, DMT, mescaline, MDMA) was used (Supplementary Information 1). ClinicalTrials.gov was also screened for ongoing studies, and we also manually screened the reference lists of all included articles and relevant reviews to identify additional eligible studies.

### Eligibility criteria

We included human studies involving adults aged 18–65 years that examined exposure to classic psychedelics (including psilocybin, LSD, DMT, and ayahuasca) or MDMA, in either clinical, naturalistic, or recreational contexts. Eligible study designs comprised randomized and non-RCT, as well as observational studies, including cohort, case–control, cross-sectional, registry-based, and prospective or retrospective longitudinal designs. Studies were required to report manic or hypomanic symptoms, manic episodes, or diagnostic transition to bipolar disorder following psychedelic or MDMA exposure, assessed through standardized clinical scales, structured diagnostic interviews, registry diagnoses, or clearly defined self-reported outcomes. Studies enrolling mixed diagnostic samples (e.g., major depressive disorder and bipolar disorder) were included only if bipolar-specific outcomes were reported separately or if individuals with bipolar disorder constituted at least 75% of the sample. We included both acute/post-acute manic or hypomanic symptoms and longer-term persistent outcomes following psychedelic exposure. When temporal detail was available, symptoms occurring during the acute phase or persisting beyond 24 h after administration were considered treatment-emergent mood outcomes, while sustained syndromes over follow-up were examined separately as persistent outcomes or diagnostic transition.

We excluded case reports and small case series, given their high susceptibility to publication and selection bias, as well as conference abstracts, editorials, commentaries, and reviews lacking original data. Studies focusing exclusively on classical psychedelic substances or MDMA were included with exclusion of cannabis, ketamine, phencyclidine, or other stimulants, as were preclinical or animal studies. Given the rarity of manic outcomes and the ethical constraints surrounding experimental exposure in bipolar populations, we adopted a broad inclusion strategy encompassing both clinical and observational designs to capture short-term affective destabilization and longer-term diagnostic transitions.

### Study screening and data extraction

All records identified through the search strategy were imported into Rayyan, a web-based tool [17] for title/abstract and full-text screening. At each stage, screening was conducted independently and in duplicate by two reviewers selected from a pool of three reviewers (ME, MS, RC). Discrepancies were resolved through discussion; when consensus could not be reached, a third reviewer adjudicated. Formal inter-rater reliability was not assessed. Data extraction was also performed independently and in duplicate by two reviewers selected from the same pool (ME, MS, RC), using a standardized Microsoft Excel spreadsheet developed a priori. Discrepancies were resolved by discussion and, when needed, by consultation with the third reviewer. Given the focus of this review, the primary outcomes were the occurrence of manic or hypomanic symptoms, manic episodes, or diagnostic transition to bipolar disorder following psychedelic or MDMA exposure. Secondary outcomes included changes in depressive symptoms, persistence or resolution of manic symptoms over time, psychiatric hospitalization, and treatment discontinuation where applicable. Additional outcomes were extracted when relevant and consistently reported across studies. When outcomes were reported in both grey literature and peer-reviewed publications, the peer-reviewed version was prioritized.

For each included study, we extracted the following information: first author, year of publication, DOI, study design, setting, sample size, participant demographics (mean age, sex/gender when available), diagnostic characteristics (e.g., bipolar I disorder, bipolar II disorder, bipolar vulnerability or family history), type of psychedelic or MDMA exposure, context of use (clinical, supervised, naturalistic, or recreational), dosing and frequency of exposure when reported, follow-up duration, outcome measures used to assess manic, hypomanic, depressive, or psychotic symptoms, timing of outcome assessment, and reported adverse events. For interventional studies, we also extracted information on preparatory and integration procedures, monitoring protocols, and discontinuation due to adverse events. Where applicable, analyses based on intention-to-treat principles were prioritized.

### Risk of bias assessment

Risk of bias was assessed independently and in duplicate by two reviewers (ME, MS), with disagreements resolved through consensus. Cross-sectional studies were appraised using the NIH Quality Assessment Tool for Observational Cohort and Cross-Sectional Studies [18].

For non-randomized and observational studies, risk of bias was assessed using the ROBINS-I tool [19], focusing on bias due to confounding, participant selection, exposure classification, deviations from intended exposure, missing data, outcome measurement, and selective reporting. For RCTs, risk of bias was assessed using the Cochrane Risk of Bias 2 (RoB 2) tool [20]. Given the predominance of naturalistic and uncontrolled designs in this literature, particular attention was paid to residual confounding, self-selection, and outcome misclassification.

Overall risk-of-bias judgments were incorporated into the interpretation of findings and informed the strength and certainty of conclusions drawn in the Discussion section. To contextualize the strength of inferences across heterogeneous study designs, the overall level of evidence supporting each results domain was graded using the Oxford Centre for Evidence-Based Medicine (OCEBM) framework [21].

### Statistical analysis

To estimate the pooled prevalence of transition to bipolar disorder following substance-related psychiatric presentations, a random-effects meta-analysis of proportions was performed. All analyses were conducted using R (version 4.4.0). Proportions were analyzed on the logit scale using the *metafor* package [22] which provides appropriate variance stabilization for prevalence outcomes, and pooled estimates were back-transformed to proportions for interpretability.

Random-effects models were fitted using the restricted maximum likelihood (REML) estimator to quantify between-study variance (τ²). Statistical heterogeneity was assessed using both τ² and the I² statistic, with I² values above 50% indicating substantial between-study heterogeneity. Given the small number of included studies, confidence intervals around pooled estimates were interpreted conservatively. Continuity corrections were not required, as all studies reported non-zero events. Funnel plots were generated for visual assessment of small-study effects; however, no formal tests for publication bias were performed due to the limited number of studies (k < 10).

## Results

### Overview of included studies

Twenty-three studies met inclusion criteria (Table 1; Supplementary Fig. 1 and Table 2), comprising three cross-sectional surveys [10, 23, 24], seven observational studies [11, 23–28], three papers on two uncontrolled clinical trials [29–31], and two RCTs [32, 33].

**Table 1:** Summary of evidence on mania and bipolar outcomes associated with psychedelic use across study designs.

Author, year	Type of study	Setting	Main findings	Mania-related outcomes	NIH, ROB2 or ROBINS-I score
Cross sectional studies					
General population					
Simonsson et al., [39]	Cross-sectional observational survey	NSDUH survey (2015–2018), US adult population (N = 2822)	Lifetime naturalistic psychedelic use was associated with more unusual visual experiences and more psychotic symptoms in the past 2 weeks only among individuals with a family history of psychotic or bipolar disorders; inverse associations observed in those without such family history.	**Follow-up duration:** Not applicable (n.a.). **Manic symptoms:** No direct rate reported. **Bipolar diagnosis (BD):** Family history only assessed. **Risk factors identified:** Family history of psychotic or bipolar disorders; recent symptom window; self-reported outcomes.	Serious risk of bias
Individuals with mood disorders					
Morton et al., [10]	Web-based cross-sectional survey	International online survey of individuals with self-reported bipolar disorder using psilocybin (N = 541); mean age = 34.1 y; 53.6% male	~32.2% reported new or increased symptoms (mania, sleep difficulties, anxiety) after psilocybin use; emergency medical service use was rare (3.3%); overall perceived benefit > harm; no difference between BD I and BD II.	**Follow-up duration:** n.a. **Manic symptoms:** 32.2% reported new or increased manic-like symptoms (composite self-report). No confirmed manic episodes reported; no hospitalization signal beyond 3.3% participants, but with no specific mention of mania. **BD:** Pre-existing self-reported diagnosis. **Risk factors identified:** no risk factors were identified, but some individuals propose that unsupervised psilocybin use major risk for a negative experience.	Serious risk of bias
Simonsson et al., [39]	Cross-sectional, genetically informative twin study	Swedish Twin Registry adolescents (N = 16,255); mean age = 15 y; 45.3% male; ~600 with schizophrenia or bipolar I history	After adjustment for other substance use, psychedelic use was associated with *lower* psychotic symptoms overall; significant interactions showed higher manic symptoms among individuals with greater genetic liability for schizophrenia or bipolar I disorder.	**Follow-up duration:** n.a. **Manic symptoms:** Association observed only in interaction analyses with genetic vulnerability; effect size reported via regression coefficients (no incidence rates). **BD:** No transition assessed; some participants had pre-existing bipolar I disorder. **Risk factors identified:** High polygenic risk for schizophrenia/bipolar I; adolescent age; shared genetic and environmental confounding.	Serious risk of bias
Observational studies					
General population					
Marrocu et al., [25]	Prospective study (naturalistic, pooled online cohorts)	Community samples; self-initiated psychedelic use; pooled 3 cohorts (N = 807); adults; mixed gender; prior diagnoses allowed	Mental health assessed pre-use and 4 weeks post-use; 16.4% met criteria for clinically meaningful deterioration (“negative responders”); among diagnostic subgroups (self-reported), 31.2% with personality disorders and 12.5% with bipolar disorders were negative responders.	**Follow-up duration:** 4 weeks post-use. **Manic symptoms:** Not reported as a specific outcome. **BD:** Self-reported pre-existing BD subgroup; 12.5% classified as “negative responders” (global deterioration, not necessarily mania). **Risk factors identified:** Pre-existing psychiatric diagnoses (personality disorders; BD subgroup flagged); self-initiated use context.	Serious risk of bias
Honk et al., [23]	Longitudinal observational	Population-based adult samples in the US and UK (N = 9732)	Psychedelic use during the study period was not generally associated with change in psychotic symptom count; effect modification: among those with personal or family history of BD, psychedelic use associated with increases in psychotic symptoms; among those with personal history of psychosis, symptoms decreased.	**Follow-up duration:** Longitudinal. **Manic symptoms:** Not reported. **BD:** Personal or family history assessed. **Risk factors identified:** Personal/family history of BD; personal history of psychosis.	Serious risk of bias
Simonsson et al., [39]	Prospective study	US adults 18–50 recruited online (N = 21,990 baseline; N = 12,345 at 2-month follow-up); 45.7% male	Naturalistic psychedelic use in illegal contexts associated with increases in psychotic and manic symptoms; increase in manic symptoms moderated by personal history of BD I (among users, those with BD I history showed greater increases).	**Follow-up duration:** 2 months. **Manic symptoms:** increase in manic symptoms for individuals with a personal history of BD I. **BD:** Personal history of BD I used as moderator; no diagnostic transition assessed. **Risk factors identified:** Illegal context of use; personal history BD I; higher-risk context features.	Serious risk of bias
Simonsson et al., [39]	Longitudinal observational cohort study	Same online US sample 18–50; baseline+2-month follow-up (N = 21,990 baseline; 12,345 follow-up)	Very small numbers with both psychedelic use during study period and personal history of schizophrenia (n = 4) or BD I (n = 22). Naturalistic use associated with increases in psychotic and manic symptom severity in adjusted models, especially in illegal contexts; effects varied by frequency, challenging experiences, and psychiatric history.	**Follow-up duration:** 2 months. **Manic symptoms:** Increase in manic symptom severity in adjusted models. **BD:** Personal history BD I subgroup (n = 22 among users). **Risk factors identified:** Illegal context; frequency of use; intensity of challenging experiences; psychiatric history (schizophrenia, BD I).	Serious risk of bias
Individuals with mood disorders					
DellaCrosse et al., [27]	Qualitative follow-up interviews	International sample of adults with bipolar disorder who self-reported psilocybin use (N = 15), recruited from a prior web survey	Participants described both perceived benefits (reduced depressive symptoms, improved emotional processing, novel perspectives, improved sleep/relaxation).	**Follow-up duration:** Qualitative follow-up after prior survey. **Manic symptoms:** Worsening of manic symptoms reported (no rates). Hospitalization due to manic symptoms reported by one participant. **BD:** All participants had pre-existing BD. **Risk factors identified:** sleep disruption, increased mania severity, hospitalization, distressing sensory experiences.	Serious risk of bias
Meyer et al., [11]	Retrospective observational naturalistic study	Community-based recreational setting; individuals with diagnosed bipolar disorder reporting recent psychedelic use (N = 105)	Psychedelic use associated with reduced days with depressive symptoms, increased symptom-free days, and reduced cannabis use; no statistically significant increase in hypomanic, manic, psychotic, or anxiety symptoms detected post-use.	**Follow-up duration:** Retrospective comparison of pre- vs post-use period. **Manic symptoms:** No statistically significant increase detected post-use. **BD:** All participants had established BD; no evidence of diagnostic worsening. **Risk factors identified:** Personal or family psychiatric history; individual vulnerability.	Serious risk of bias
Vale et al., [28] *(follow-up of Meyer et al*., [11]*)*	Longitudinal observational study	Community-based naturalistic psychedelic use; adults assessed at baseline and follow-up (N = 203) using self-reported manic and psychotic symptom measures	No consistent increase in manic or psychotic symptoms at the population level; symptom changes moderated by individual vulnerability; higher increases observed among those with personal or family history of mood or psychotic disorders; most changes were transient and not associated with severe outcomes.	**Follow-up duration:** Longitudinal follow-up of 4 months. **Manic symptoms:** No population-level increase. **BD:** Participants included individuals with BD; no diagnostic transition assessed. **Risk factors identified:** Personal or family psychiatric history; individual vulnerability;	Serious risk of bias
CT					
Individuals with mood disorders					
Goodwin et al., [32]	Randomized, double-blind, dose-finding controlled trial (Phase 2b)	Multicenter international outpatient trial (Europe & North America); treatment-resistant major depressive disorder	Psilocybin 25 mg plus psychological support produced greater short-term reductions in depressive symptoms compared with 1 mg control; effects attenuated over follow-up.	**Follow-up duration:** up to 12 weeks. **Manic/hypomanic episodes:** None reported, however, 4 participants ( ≈ 5%) experienced transient euphoric or dysphoric mood between 2 days and 3 weeks post-administration. **BD:** no diagnostic transition assessed. **Risk factors identified:** no risk related to bipolar disorders was proposed	Some concerns
Bogenschutz et al., [33]	Randomized, double-blind, active-placebo–controlled trial	Academic clinical research centers (USA); outpatient setting; alcohol use disorder	Psilocybin (25–40 mg/70 kg) combined with psychotherapy significantly reduced alcohol consumption and increased abstinent days compared with diphenhydramine control.	**Follow-up duration:** up to 32 weeks. **Manic/hypomanic episodes:** None reported, but one case of dysphoric mood is reported **during follow-up**. **BD:** no diagnostic transition assessed. **Risk factors identified:** no risk related to bipolar disorders was proposed	Low risk
Aaronson et al., [16]	Non-randomized open-label trial	Single centre (US, Sheppard Pratt Health System); adults 18–65 with treatment-resistant bipolar II depression; N = 15	Single 25 mg synthetic psilocybin with psychotherapy: large antidepressant effect (MADRS mean change −24 at 3 weeks; Cohen’s d = 4.08). No significant increase in YMRS or suicidality.	**Follow-up duration:** 3 weeks primary endpoint. **Manic symptoms:** YMRS did not significantly increase from baseline. **BD:** no diagnostic transition assessed. **Risk factors identified:** Not specified.	Moderate risk of bias
Rosenblat et al., [30]	Open-label psilocybin-assisted psychotherapy study	Adults with treatment-resistant depression, including some with bipolar II disorder; N = 29	Repeated 25 mg psilocybin+psychotherapy associated with significant and sustained depression reduction over 6 months; effects maintained across repeated dosing.	**Follow-up duration:** 6 months. **Manic symptoms:** YMRS did not significantly increase from baseline. **BD:** Subset with BD II included, no diagnostic transition assessed. **Risk factors identified:** Not specified.	Serious risk of bias
Meshkat et al., [31] *(follow-up of Rosenblat et al*., [30]*)*	Non-randomized open-label trial	Adults with treatment-resistant depression in bipolar II disorder; N = 15; 1–2 sessions of 25 mg psilocybin+psychotherapy	MADRS decreased ( ≈ 32.5 baseline → ≈20 week 2) and remained ≈21 at 6 months. YMRS remained stable ( ~ 1). No emergent mania, hypomania, or psychosis during follow-up.	**Follow-up duration:** 6 months. **Manic symptoms:** YMRS stable at ~1 across follow-up. **BD:** no transition assessed. **Risk factors identified:** Not specified.	Moderate to serious risk of bias
Registry-based cohort studies					
Individuals with substance use disorder					
Starzer et al., [36]	Population-based registry cohort study	Denmark; national registers (Psychiatric Central Research Register & Civil Registration System); substance-induced psychosis (N = 6788)	32.2% converted to schizophrenia or bipolar disorder (95% CI 29.7–34.9). Highest conversion in cannabis-induced psychosis (47.4%, 95% CI 42.7–52.3). Conversion to bipolar disorder was 5.3% (361/6788); half occurred within ~4.4 years after index psychosis.	**Follow-up duration:** Longitudinal, 20 years duration. **Manic symptoms:** Not assessed, however conversion was reported. **BD:** 5.3% (361/6788) converted to BD; time-to-conversion: ~50% within ~4.4 years. **Risk factors identified:** Younger age, self-harm post-index; substance category strongly associated with overall conversion (in particular cannabis-induced psychosis) but no hallucinogen-specific effect.	Moderate risk of bias
Rognli et al., [35]	Population-based registry cohort study	Norway; national patient registry; substance-induced psychosis (N = 3187)	6-year conversion: 4.5% (95% CI 3.6–5.5) to BD; 27.6% (95% CI 25.6–29.7) to schizophrenia-spectrum disorder.	**Follow-up duration:** Longitudinal, 5 years duration. **Manic symptoms:** Not assessed (registry). **BD:** 4.5% at 6 years (95% CI 3.6–5.5). **Risk factors identified:** Younger age, polysubstance use, however no specific risk related to hallucinogens was reported.	Moderate risk of bias
Myran et al., [40]	Population-based registry cohort study	Ontario (Canada) health databases; individuals receiving hospital-based care for hallucinogen-related conditions with longitudinal follow-up (N = 7285)	Within 3 years after hallucinogen-related acute care, 1.43% (n = 104) had an incident mania episode requiring acute care vs 0.06% (n = 41) in matched general population. In acute/outpatient hallucinogen-related care cohort, 5-year BD conversion was 8.55% (579/6772). Risk highest in first year, attenuated over time; interpretation favors combined substance-related effects+underlying vulnerability/prodrome rather than typical recreational/therapeutic use.	**Follow-up duration:** Longitudinal, 14 years duration. **Manic symptoms:** Mania was reported: 1.43% (104/7285) incident acute-care mania within 3 years. **BD**: 8.55% (579/6772) new BD diagnosis within 5 years among those with acute or outpatient hallucinogen-related care. **Risk factors identified:** Temporal proximity (highest risk in year 1); likely baseline vulnerability/prodromal illness; care-setting severity (hospital/outpatient hallucinogen-related presentation) as a marker of risk.	Moderate risk of bias
Individuals with mood disorders					
Wirowski et al., [34]	Registry-based cohort study	Population-based cohort of adults aged 18–60 with major depressive disorder (N = 468); lifetime substance use assessed	Overall conversion rate from MDD to bipolar disorder was 12.4%. Among those who converted, 2.3% reported lifetime hallucinogen use. Of individuals with lifetime hallucinogen exposure (n ≈ 10), only 1 transitioned to bipolar disorder over follow-up.	**Follow-up duration:** Long-term registry follow-up, of 6 year duration. **Manic symptoms:** Not reported. **BD:** Conversion rate 12.4% overall; among hallucinogen-exposed individuals, ~10% (1/10) converted, based on very small, exposed subgroup. **Risk factors identified:** no specific risk factors identified for psychedelics.	Moderate to serious risk of bias

Risk of bias and study quality were assessed using design-appropriate tools. Observational studies were evaluated using the NIH Quality Assessment Tool (Good/Fair/Poor). Non-randomized studies were assessed with ROBINS-I (Low/Moderate/Serious/Critical). Ratings reflect methodological rigor and internal validity and do not imply causality. For randomized studies, ROB2 was used. Overall risk reflects the highest concern across domains; some concerns mainly due to likely functional unblinding and/or attrition, otherwise low risk when all domains were judged low risk. Tables for risk of bias are available in the supplements

**Table 2:** Ongoing and recent psychedelic-related clinical trials in Bipolar II disorder.

NCT ID	Trial title	Intervention	Design & sample	Primary objectives	Current status
NCT05065294	Psilocybin Therapy for Depression in Bipolar II Disorder	Psilocybin (oral) 10–25 mg, single administration, with psychological support and monitoring	Open-label pilot study; small sample (14 participants) with bipolar II depression	Assess safety, tolerability, and feasibility of psilocybin-assisted therapy in bipolar II depression	Ongoing (not recruiting) – active follow-up
NCT05839509	Phase 2 Clinical Trial of GH001 in Bipolar II Disorder	GH001 (inhaled mebufotenin / 5-MeO-DMT), individualized dosing regimen (1–3 doses in a single day)	Multicenter, open-label, single-arm Phase 2 trial; planned 15 participants	Evaluate safety and feasibility of GH001 in bipolar II depression	Terminated (Clinical hold by FDA- 6/15 patient enrolled with no adverse event reported)
NCT06506019	Neurobiological Effects of Psilocybin in Treatment-Resistant Bipolar Depression (Psilo-BD)	Psilocybin (oral) 25 mg single dose+preparatory, monitoring, and integration psychotherapy (PAP)	Open-label, single-arm, proof-of-concept study in treatment-resistant bipolar depression	Characterize neurobiological mechanisms of psilocybin using fMRI	Ongoing
NCT06706232	Acceptability & Safety of Two Sequential Doses of Psilocybin in Bipolar Disorder II Depression and Suicidality	Psilocybin (oral) up to two sequential doses of 25 mg + enhanced therapeutic support	Open-label Phase 2 study; 10 participants	Assess safety, acceptability, and effects on suicidality in bipolar II depression	Recruiting

Four additional article on the prevalence of transition to bipolar disorders following hallucinogens use were identified [34–37]. One literature review on 128 case reports of drug-induced mania [38], was not retained, considering our inclusion criteria. Furthermore, three ongoing feasibility trials investigating psilocybin-based interventions in bipolar II disorder were also identified (NCT05065294; NCT06506019; NCT06706232), as one phase II DMT study with treatment-resistant bipolar type II individuals (NCT05839509).

Overall, except for some short-term feasibility trials, the evidence base was dominated by naturalistic and self-reported data, with most studies rated as having serious risk of bias under ROBINS-I or equivalent frameworks (Table 1; Supplementary Table 3).

### Studies in the general population and individuals with bipolar vulnerability

Several large population-based studies examined associations between psychedelic use and manic or psychotic symptoms in the general population, with particular attention to the moderating role of bipolar or psychosis vulnerability. Overall, these studies did not support a uniform association between psychedelic exposure and manic symptoms at the population level. Rather, risk appeared to be concentrated among individuals with elevated genetic, familial, or personal vulnerability to bipolar or psychotic disorders.

Using a genetically informative Swedish twin cohort, Simonsson et al. [39] found no overall association between lifetime psychedelic use and manic symptoms at the population level. However, a significant interaction was observed whereby individuals with higher polygenic risk scores for schizophrenia or bipolar I disorder exhibited greater manic symptom severity following psychedelic exposure, whereas no such effect was observed in individuals with lower genetic liability.

Similarly, in large U.S. population samples, Simonsson et al. [39] reported that lifetime psychedelic use was associated with increased psychotic-like and unusual perceptual experiences among individuals with a family history of psychotic or bipolar disorders, whereas no such association, or even lower symptom burden, was observed in those without such familial vulnerability. Honk et al. [23] likewise found no overall association between psychedelic use and psychotic symptoms, but identified increased symptom counts among participants reporting a personal or family history of bipolar disorder.

Importantly, through these studies, the reported manic or psychotic symptoms were generally subsyndromal and did not provide evidence of sustained mood episodes or diagnostic transition. Taken together, these findings support a diathesis–stress interpretation, in which psychedelic exposure does not appear to increase manic risk uniformly in the general population, but may be associated with greater affective or psychotic symptom expression in individuals with underlying bipolar-spectrum or psychosis vulnerability.

I would also slightly adjust the Discussion afterward so it matches the new structure, replacing wording like “healthy participants and the general population” plus “individuals with bipolar vulnerability” with one single evidence domain.

### Studies in individuals with diagnosed bipolar disorder or self-reported bipolar disorder

Multiple studies investigated psychedelic-associated manic symptoms among individuals with a diagnosed or self-reported bipolar disorder.

Morton et al. [10] conducted a large international web-based survey of 541 individuals with self-reported bipolar disorder who had used psilocybin. Approximately 32.2% reported new or worsened symptoms—including manic symptoms, sleep disturbance, or anxiety—during the week following psilocybin use. Emergency medical service utilization was rare (3.3%), and most respondents rated psilocybin as beneficial overall. No significant differences were observed between bipolar I and bipolar II subgroups.

Marrocu et al. [25] pooled data from three prospective naturalistic cohorts assessing mental health before and four weeks after psychedelic use. While most participants demonstrated improvement in well-being, 16.4% met criteria for clinically meaningful deterioration. Among participants with self-reported bipolar disorder, 12.5% were classified as “negative responders,” indicating worsening of psychiatric symptoms following psychedelic exposure.

In contrast, Meyer et al. [11] conducted a retrospective within-subject pre–post analysis in 105 individuals with bipolar disorder. Psychedelic use was associated with reductions in depressive symptoms, decreased cannabis use, and increased symptom-free days, with no statistically significant increase in manic, psychotic, or anxiety symptoms over a three-month follow-up period.

Longitudinal analyses by Simonsson et al. (2025) further demonstrated that naturalistic psychedelic use—particularly in illegal or unsupervised contexts—was associated with increases in manic and psychotic symptoms over two months, with stronger effects among individuals with bipolar I disorder. Symptom severity was moderated by frequency of use and intensity of challenging experiences.

Overall, studies in diagnosed bipolar populations suggest a heterogeneous risk profile, with transient manic or hypomanic symptoms occurring in a minority of individuals, particularly those with bipolar I disorder and exposure occurring in unsupervised or high-risk contexts.

### Clinical studies of psychedelic-assisted psychotherapy

The few published controlled clinical studies evaluating psychedelic-assisted psychotherapy in bipolar depression have consistently reported a relatively favorable safety profile with respect to treatment-emergent manic or hypomanic symptoms.

Aaronson et al. [16] reported large antidepressant effects following a single 25 mg dose of psilocybin administered with psychotherapy in individuals with bipolar II depression, with no increase in Young Mania Rating Scale scores and no emergent suicidality.

Rosenblat et al. [30] conducted a clinical trial in treatment-resistant depression that included a subset of individuals with bipolar II disorder. Sustained antidepressant effects were observed across repeated dosing, with no systematic emergence of manic or hypomanic symptoms during follow-up. Similarly, Meshkat et al. [31] reported significant improvements in depressive symptoms following psychedelic-assisted psychotherapy, with stable YMRS scores and no manic or psychotic exacerbations.

Across all uncontrolled clinical trials, no persistent manic episodes were observed. However, sample sizes were small, follow-up durations were limited, and exclusion criteria were stringent, limiting generalizability to broader bipolar populations.

However, we identified two RCTs on individuals with major depressive disorders, and alcohol use disorders [32, 33] that systematically monitored participants’ mood for several weeks following psychedelic administration. In the study by Goodwin et al., participants were followed for up to 12 weeks post-treatment, during which 4 individuals (5%) experienced transient euphoric or dysphoric mood states occurring between 2 days and 3 weeks after administration. Other participants even experienced anxiety and depressive states (4(5%) and 3(4%) respectively). In contrast, Bogenschutz and colleagues monitored participants for up to 32 weeks following treatment and reported a single case of dysphoric mood during the follow-up period. Notably, many RCTs did not provide detailed longitudinal reporting of post-acute mood changes following psychedelic administration. Overall, as reported in one of our previous publications [5], the pooled proportion of participants experiencing ‘euphoric’ mood states in RCTs is approximately 5.8%.

### Prevalence of transition to bipolar disorder following psychedelic exposure

Four registry-based cohort studies examined the prevalence of transition to bipolar disorder among individuals with substance-related psychopathology. Starzer et al. [36] reported that among individuals that received a diagnosis of hallucinogen-induced psychosis, 5.26%(6/114) transitioned to bipolar disorder over follow-up, with bipolar transitions typically occurring within five years follow-up window.

Rognli et al. [35] reported a six-year transition rate of 5.80% to bipolar disorder following hallucinogen-induced psychosis in a Norwegian national cohort occurring over six years. Wirowski et al. [34] examined transition from major depressive disorder to bipolar disorder among individuals with lifetime use of hallucinogens and found a 12.4% transition rate overall and a 10% transition rate among individuals who had a lifetime hallucinogen use, occurring over six years.

Myran et al. [40] reported a 3-year incident rate of mania requiring acute care among those who had emergency visits for hallucinogen use was approximately 1.43%, compared to 0.06% in the matched general population. Myran et al. [40] reported a five-year cumulative incidence of 3.13% for bipolar disorder among individuals who received acute care for hallucinogen-related conditions, compared with 0.86% in matched population controls, indicating an elevated prevalence of subsequent transition to bipolar disorder diagnosis following severe hallucinogen-associated presentations. This prevalence was even higher, at 8.55%, when considering acute care and outpatient setting. Authors found that the transition prevalence was highest within the first year following hospital presentation and attenuated over longer follow-up.

**Fig. 1:**
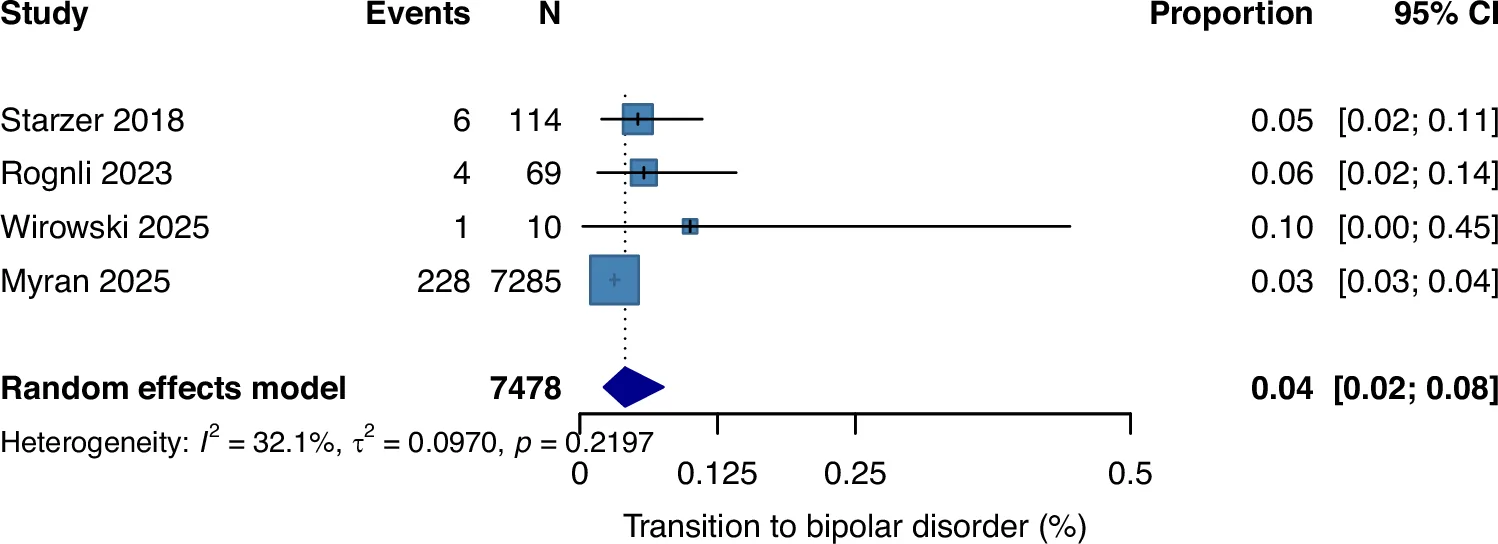
Forest plot of the proportion of transition to bipolar disorder following hallucinogen-induced psychosis or mood disorder (random-effects logit model). Squares are proportional to inverse-variance random-effects weights estimated on the logit scale. The pooled estimate was largely driven by the population-based registry study by Myran et al., [40] (88% of the total random-effects weight), whereas Starzer et al., [36] (6%), Rognli et al., [35] (5%), and Wirowski et al., [34] (1%, descriptive only) contributed limited statistical weight due to small event counts and wide confidence intervals. Between-study heterogeneity was low to moderate (I² = 32.1%, τ² ≈ 0.10).

We conducted an exploratory meta-analysis on the prevalence of transition from hallucinogens-induced mood/psychosis disorders to a diagnosis of bipolar disorder within a 5–6-year time-window (Fig. 1). Across all studies, the pooled prevalence of transition to bipolar disorder was estimated at approximately 4%(95%CI: 2–8%; p < 0.001; N = 7478), in presence of a low-to-moderate heterogeneity (I² = 32.1%).

Following the visual inspection of the correspondent funnel plot (Supplementary Fig. 2) and considering that the consequent weight of the Myran et al. [40] study, we excluded this study in a subgroup analysis (Supplementary Fig. 2). The pooled prevalence of transition to bipolar disorder was similar 6%(95%CI: 3–10%; p < 0.001; N = 193), in absence of heterogeneity (I² = 0%) (Supplementary Fig. 3). Overall, these data support a small statistically significant association between hallucinogen exposure and diagnostic transition to bipolar disorder (4–6% prevalence). However, considering the heterogeneity in study designs, these findings should be interpreted cautiously and viewed as hypothesis-generating these registry-based.

### Ongoing and registered clinical trials

Four ongoing or registered clinical trials are currently evaluating psychedelic interventions in bipolar II disorder, including psilocybin-assisted psychotherapy (NCT05065294; NCT06506019; NCT06706232) and a phase 2 trial with GH001(5-MeO-DMT) (NCT05839509)(Table 2). All are open-label or single-arm studies primarily designed to assess feasibility, safety, and neurobiological mechanisms rather than long-term manic risk. However, NCT05839509 was terminated due to regulatory and development strategy considerations.

## Discussion

This systematic review synthesized evidence from clinical trials, observational studies, and registry-based cohorts to examine the prevalence of manic symptoms and diagnostic transition following psychedelic exposure in individuals with bipolar disorder or bipolar vulnerability. When interpreted in light of the evidence grading, a consistent domain-specific pattern emerges (Table 3). Moderate-quality evidence (Level 2b) from population-based and genetically informed cohorts indicates that psychedelic exposure is not associated with increased manic symptoms at the population level, with risk emerging primarily among individuals with elevated genetic or familial liability. In contrast, evidence regarding individuals with diagnosed bipolar disorder—particularly bipolar I disorder—is derived mainly from naturalistic and self-reported studies (Level 3b), which suggest a modest incidence of transient manic or hypomanic symptoms, especially in unsupervised or illegal contexts. Importantly, serious outcomes such as hospitalization or sustained diagnostic progression were uncommon across studies, although their true incidence remains uncertain given methodological limitations.

**Table 3:** Synthesis of the evidence.

Clinical domain/setting (Results section)	Study design	Domain-level conclusion (synthesized)	OCEBM level	Strength interpretation
**General populations, naturalistic setting**				
Healthy participants / general population	Large population surveys; genetically informed cohort (twin study); population cohorts	Psychedelic exposure is not associated with increased manic symptoms at the population level; risk emerges only in individuals with elevated genetic or familial vulnerability.	Level 2b	**Moderate evidence** from well-designed cohort and population studies with consistent moderation effects
**At-risk population, naturalistic**				
Bipolar vulnerability (genetic, familial, subthreshold)	Population cohorts with stratification by polygenic risk scores or family history; observational studies	Psychedelics may precipitate transient manic or psychotic symptoms in individuals with latent bipolar vulnerability, but do not generally induce sustained syndromal mania or diagnostic transition.	Level 2b–3b	**Moderate-to-low evidence**; consistent signal across studies but limited by observational design and residual confounding
**Bipolar disorder, naturalistic setting**				
Bipolar disorder	Cross-sectional surveys; longitudinal naturalistic cohorts; retrospective within-subject analyses	A minority of individuals with bipolar disorder experience transient manic or hypomanic symptoms after naturalistic psychedelic use, particularly in bipolar I disorder and unsupervised contexts; persistent mania is uncommon.	Level 3b	**Low-to-moderate evidence** from non-randomized observational studies; heterogeneity and self-report bias present
**Substance use disorder, naturalistic**				
Psychedelic-induced disorder	National registry-based cohort studies; longitudinal follow-up	Taken together, registry-based data do not support a low-magnitude association (4%) between hallucinogen exposure and diagnostic transition to bipolar disorder.	Level 2b	**Moderate evidence** from large, longitudinal registry studies with objective outcomes
**Mental disorders, experimental settings**				
Clinical psychedelic-assisted psychotherapy	Uncontrolled clinical trials with systematic monitoring and controlled clinical trials	In carefully screened and supervised clinical settings with individuals with bipolar disorders, psychedelic-assisted psychotherapy is not associated with treatment-emergent or persistent mania. However, in RCTs with major depressive disorders, treatment-emergent euphoria and dysphoria are found (5.8%).	Level 2b	**Moderate evidence** from prospective interventional studies, limited by small samples and lack of controls

Despite heterogeneity in study design and outcome assessment, the overall evidence base—graded predominantly at Levels 2b–3b—does not support a strong or generalizable causal relationship between psychedelic use and the induction of bipolar disorder. Rather, the data suggests that manic symptoms, when they occur, are typically transient, context-dependent, and concentrated in high-risk subgroups. This distinction between short-term affective destabilization and persistent bipolar illness is critical, particularly given diagnostic criteria requiring symptom persistence beyond substance effects. Taken together, and consistent with the evidence hierarchy presented in Table 3, the reviewed literature supports a nuanced interpretation in which psychedelics may confer situational risk in vulnerable individuals while appearing relatively safe with respect to manic outcomes in carefully screened and monitored clinical settings.

### Short-term vs. persistent effects

Across the included studies, manic or hypomanic symptoms associated with psychedelic exposure were predominantly acute and time-limited, typically emerging within 24–72 h following use and resolving over days to weeks. Cross-sectional and longitudinal observational studies consistently described symptom exacerbations occurring in close temporal proximity to psychedelic ingestion, most often characterized by transient increases in mood elevation, reduced need for sleep, or behavioral activation (Morton et al., [10]). Importantly, in prospective and retrospective observational cohorts with follow-up extending from several weeks to months, no study demonstrated evidence of persistent or progressive manic syndromes attributable solely to psychedelic exposure [11, 25]. In controlled therapeutic contexts, including multiple uncontrolled clinical trials of psilocybin-assisted psychotherapy in bipolar II disorder, systematic monitoring using standardized scales, showed transient euphoric, dysphoric symptoms for individuals with depressive disorders [32, 33], while for individuals with bipolar disorders potential transient activation resolved without pharmacological intervention [16, 31].

When manic symptoms persisted beyond the expected window of substance effects, the clinical course was more consistent with underlying bipolar disorder unmasked or precipitated by psychedelic exposure, rather than with a substance-induced bipolar disorder as defined by DSM-5 criteria. This interpretation is further supported by registry-based cohort studies of substance-induced psychosis, which demonstrated that diagnostic transitions to bipolar disorder typically occurred over years, not acutely, and were associated with established vulnerability factors rather than with hallucinogen exposure itself [35, 36]. Collectively, these findings suggest that psychedelics may precipitate short-term affective destabilization in vulnerable individuals, but do not appear to induce persistent bipolar illness in the absence of pre-existing liability.

### Transition to bipolar diagnosis

Our systematic review proposes that the transition to a formal bipolar diagnosis following psychedelic-associated manic symptoms was difficult to establish, with long-term outcome data remaining limited. Observational and registry-based cohort studies examining diagnostic transitions after substance-related psychopathology indicate that conversion to bipolar disorder, when it occurs, typically unfolds over years rather than acutely, and is strongly influenced by pre-existing vulnerability factors rather than by hallucinogen exposure itself [35, 36]. In contrast, the only large hallucinogen-specific population cohort to date reported a low absolute prevalence of transition to bipolar disorder (3–6%), comparable to background rates and not exceeding prevalences observed with other substances [37, 40].

In the only longitudinal cohort to explicitly report hallucinogen exposure in patients with unipolar disorders, lifetime use was limited (17.8%) and only a single hallucinogen-exposed individual transitioned to bipolar disorder, precluding any inference of substance-specific risk [34]. For Starzer and Rognli studies, 4 and 3.8% for amphetamine-induced psychosis, 5 and 4.9% for cannabis-induced psychosis were found respectively. These risks are secondary to schizophrenia-spectrum transitions due to cannabis and amphetamines (around 30%) [41, 42], and suggest that the transition risk is not substance-specific, but rather reflect shared vulnerability factors (affective instability, bipolar-spectrum vulnerability, stress-diathesis mechanisms). Furthermore, Sabé et al. proposed a 13.1% of prevalence of transition from psychedelic-induced psychosis to schizophrenia, which advocate for a lower risk of transition when considering serotoninergic psychedelics [4].

Within cross-sectional and prospective observational studies of psychedelic use among individuals with self-reported bipolar disorder, some participants reported manic episodes temporally associated with psychedelic exposure; however, no study provided systematic evidence that these episodes resulted in sustained bipolar illness attributable to psychedelics [10, 25, 26, 43]. Notably, in therapeutic and research contexts, individuals with bipolar II disorder who experienced transient activation or hypomanic symptoms frequently remained episode-free during follow-up, with no progression to bipolar I disorder or recurrent mania [16, 31].

According to DSM-5 criteria, a manic episode that persists beyond the expected physiological effects of a substance is sufficient to warrant a diagnosis of bipolar I disorder. However, the available evidence suggests that such sustained episodes following psychedelic exposure are rare, and when diagnosed, are more plausibly interpreted as manifestations of previously unrecognized bipolar disorder unmasked by an acute affective stressor, rather than as true substance-induced bipolar disorder. Consequently, while isolated cases of diagnostic transition have been reported [38], the current literature does not support a robust causal link between psychedelic exposure and durable bipolar disorder onset in the absence of underlying susceptibility.

### Risk factors and prominent moderators identified

Our findings suggest that the emergence of manic or hypomanic symptoms following psychedelic exposure appears to be strongly moderated by pre-existing vulnerability factors rather than by psychedelic use alone. An interpretive possibility is that psychedelic use may, in some cases, represent a behavioral manifestation of emerging hypomania or mania rather than a purely exogenous trigger. This is particularly relevant in bipolar disorder, where prodromal or early syndromal phases may include increased energy, impulsivity, risk-taking, reduced sleep, and behavioral disinhibition, potentially complicating causal attribution in naturalistic studies [44]. The most consistently identified moderator was a personal history of bipolar disorder, particularly bipolar I disorder, with multiple cross-sectional and longitudinal studies reporting greater manic symptom expression among individuals with established bipolar diagnoses following naturalistic psychedelic use [10, 24, 26]. Familial and genetic vulnerability further amplified risk: in a genetically informed twin study, psychedelic use was associated with increased manic symptoms only among individuals with higher polygenic risk scores for schizophrenia or bipolar I disorder, whereas no such association was observed in those with lower genetic liability [39].

Context of use emerged as another critical moderator. Longitudinal observational data indicated that psychedelic use occurring in illegal or unsupervised settings was associated with increases in manic and psychotic symptom severity, particularly among participants with bipolar I disorder or other severe psychiatric histories [23, 26]. In contrast, structured therapeutic contexts, including uncontrolled clinical trials of psilocybin-assisted psychotherapy in bipolar II depression, consistently showed no evidence of emergent mania or hypomania, despite robust antidepressant effects [16, 31].

Co-exposures and clinical state factors were frequently implicated in adverse outcomes. Several observational studies highlighted the role of poly-substance use, particularly stimulants and antidepressants, as well as sleep deprivation and psychosocial stress, as proximal contributors to manic symptom emergence following psychedelic exposure [10, 25]. Importantly, large registry-based cohort studies examining diagnostic transitions following substance-induced psychosis found no hallucinogen-specific signal for conversion to bipolar disorder, with risk largely driven by younger age, self-harm, and non-psychedelic substances such as cannabis or stimulants [35, 36].

Taken together, the available evidence supports a diathesis–stress model, in which psychedelics may act as non-specific affective or neurobiological stressors capable of precipitating manic symptoms primarily in individuals with pre-existing bipolar liability, high genetic risk, or destabilizing contextual factors, rather than as independent causal agents of bipolar disorder.

### Theoretical framework: psychedelics as precipitants, sensitizers, or inducers of bipolarity

A key unresolved question is whether repeated psychedelic exposure could contribute to a kindling- or sensitization-like process, whereby the threshold for affective episodes becomes progressively lower in vulnerable individuals. This possibility is conceptually related to the affective kindling model described in antidepressant-associated bipolarity (sometimes referred to as “Type III” bipolar disorder), in which repeated pharmacological perturbations have been hypothesized to increase affective instability over time rather than induce bipolar disorder de novo [45].

From a theoretical perspective, psychedelics could plausibly participate in such a process. Acute serotonergic and downstream glutamatergic perturbations, together with sleep disruption, emotional intensity, and stress-related arousal, may transiently destabilize affective regulation. In individuals with latent bipolar vulnerability, repeated exposures might hypothetically lower the threshold for subsequent manic or depressive episodes through sensitization mechanisms. This framework has been discussed in narrative and conceptual reviews of psychedelic-related adverse events, which note phenomenological similarities between psychedelic-associated manic reactions and antidepressant-induced mood switching [8, 46].

However, no empirical study included in this systematic review directly examined a kindling or sensitization trajectory. Available longitudinal observational studies did not show progressive worsening of bipolar course with repeated psychedelic use, nor did registry-based cohorts identify hallucinogens as clear drivers of diagnostic transition or recurrent affective cycling. Similarly, uncontrolled clinical trials involving one or two supervised psychedelic sessions did not provide evidence of cumulative manic risk or increasing affective instability over follow-up. When manic symptoms were reported, they were generally acute, temporally related to exposure, and non-recurrent, which does not support a clearly established kindling process.

Thus, while a kindling or sensitization model is well supported in antidepressant-induced bipolarity and with certain pro-dopaminergic agents [47], no comparable empirical evidence currently demonstrates progressive affective sensitization following psychedelic exposure. In the available literature, psychedelic-associated manic symptoms appear more often episodic and exposure-linked than cumulative. At present, this supports only a speculative sensitization hypothesis rather than a demonstrated kindling mechanism. This limitation is also consistent with the type and duration of the studies currently available. Longitudinal studies specifically designed to assess repeated exposure, episode recurrence, and temporal changes in affective threshold are needed to evaluate this hypothesis more directly.

### Strengths and limitations

This review integrates evidence from diverse study designs, including large registry-based cohorts, observational studies, and clinical trials, allowing triangulation across clinical and population settings and providing insight into both short-term manic symptoms and long-term diagnostic transitions. The inclusion of national registry studies strengthens conclusions regarding rare outcomes, while therapeutic trials contribute systematically monitored safety data.

However, the overall evidence base is limited by substantial risk of bias. Most studies were observational, uncontrolled, and cross-sectional, limiting causal inference. Self-selection, recall bias, exposure misclassification, and residual confounding (e.g., co-substance use, antidepressants, sleep deprivation) were common. Follow-up durations were often short, and individuals with severe adverse outcomes may have been underrepresented. Only few studies were eligible for quantitative synthesis because longitudinal data specifically examining bipolar diagnostic transition after hallucinogen exposure remain scarce, and most transition studies do not report hallucinogen-specific outcomes in a form suitable for pooling. An additional limitation concerns the search strategy. Because searches did not include full-text records, some studies may have been missed if bipolar or manic outcomes were not reported in the title, abstract, keywords, or indexing terms. Publication bias is also likely. Therapeutic studies may under detect mood-related disturbances due to strict exclusion criteria and intensive monitoring, while survey and case-based reports may overemphasize adverse events. Psychedelic trials are generally short, although very carefully monitored [48]. Consequently, although the available evidence does not support a strong causal link between psychedelic use and persistent bipolar disorder, confidence in precise risk estimates remains limited, underscoring the need for prospective, well-controlled studies with systematic adverse-event reporting.

## Conclusion

Across the available evidence, psychedelic use is associated with a low but clinically relevant risk of manic or hypomanic symptoms in individuals with bipolar vulnerability, particularly in naturalistic or unsupervised settings. In contrast, the few controlled clinical studies conducted in carefully screened bipolar II populations have not demonstrated treatment-emergent or persistent manic episodes, suggesting that structured therapeutic contexts substantially mitigate this risk. Nevertheless, transient mood disturbance, including euphoric or dysphoric state, have been reported in RCTs conducted in populations with major depressive disorder.

The existing literature does not support the induction of chronic mania or de novo bipolar disorder following psychedelic exposure. When manic symptoms do occur, they are typically acute and self-limited and are more consistent with affective destabilization or the unmasking of latent bipolar vulnerability than with DSM-5 substance-induced bipolar disorder. Importantly, the absence of evidence for long-term harm should not be interpreted as evidence of long-term safety.

While psychedelic-assisted psychotherapy shows short-term promise for treatment-resistant bipolar depression, long-term psychiatric outcomes—including cumulative effects, sensitization processes, and dose–response relationships—remain insufficiently characterized. Accordingly, screening procedures should emphasize dimensional assessments of affective instability, symptom trajectory, and longitudinal stability rather than categorical diagnostic exclusion alone, and should be embedded within rigorous monitoring frameworks and prospective longitudinal controlled studies prior to broader clinical implementation.

## Supplementary information

Supplementary material Psychedelic-induced hypomania and mania: A systematic review and meta-analysis
