# Supplementary material for: Psychedelic-induced hypomania and mania: a systematic review and meta-analysis
Source: Mol Psychiatry. 2026 May 29;31(10):5749–63. doi: 10.1038/s41380-026-03657-6 (PMC13658036; doi:10.1038/s41380-026-03657-6)
Supplement: Supplementary file 1 [file 41380_2026_3657_MOESM1_ESM.docx]

**Supplementary material**

**Psychedelic-induced hypomania and mania: A systematic review and meta-analysis**

Mickael Eskinazi, Rayan Nasserdine, Romane M Cusin, Pantelis Baniotoupoulos, Luigi F Saccaro, Marco De Pieri, Tamara Corino, Federico Seragnoli, Jean-Francois Briefer, Tatiana Aboulafia Brakha, Hélène Richard Lepouriel, Louise Penzenstadler, Kerem Böge, Matthias Kirchner, Daniele Zullino, Mikkel Højlund, Jacopo Sapienza, Marta Bosia, Ana Catalan, Eduard Vieta, Marco Solmi, Michel Sabé

**Supplementary Table 1.** Phenomenology of psychedelic-induced mood symptoms.

**Supplementary Information 1.** Search terms used for our systematic review.

**Supplementary Figure 1.** Prisma 2020 Flow diagram.

**Supplementary Table 2.** Excluded studies from our systematic review, when assessed for eligibility.

**Supplementary Table 3.** Risk of bias assessment using ROBINS-I, ROB2 or NIH.

**Supplementary Figure 2.** Funnel plot of the prevalence meta-analysis with all included studies.

**Supplementary Figure 3.** Forest plot of the proportion of transition to bipolar disorder following hallucinogen-induced psychosis or mood disorder (random-effects logit model) with exclusion of Myran et al. 2025 study.

**Supplementary Table 1.** Phenomenology of psychedelic-induced mood symptoms.

| **Term** | **Definition** | **Diagnostic criteria/ Time Frame** | **DSM/CIM reference** |
| --- | --- | --- | --- |
| **Transient symptoms** | | | |
| Trip-related euphoria | Euphoric or elevated mood limited to the duration of a psychedelic trip that may mimic—but does not meet criteria for—hypomania or mania. | Appears during acute intoxication; typically resolves within hours. Lack of sustained behavioral impairment differentiates it from mania. | Not a DSM-5 diagnostic category. |
| Persistent psychedelic Effects | Psychological or emotional effects of psychedelics (e.g., mood elevation, spiritual ideation) that persist beyond the acute intoxication phase (>1 month). | Emotional or cognitive effects persist >1-month post-trip. Not necessarily pathological but may interfere with functioning. | Not in DSM-5; persistent effects may mimic dysthymia or adjustment disorder. |
| **Persistent symptoms – declaring a hypomanic, manic or mixed episode** | | | |
| Psychedelic-Induced mania/hypomania | Onset of mania or hypomania following the immediate use of serotonergic psychedelics or MDMA.  Known as ‘‘treatment-emergent mania/hypomania’’ in the context of a psychedelic-assisted therapy. | Symptoms emerge within hours to days after use; typically last days to weeks. Often resolves with appropriate treatment. | Parallels DSM-5 criteria for Substance/Medication-Induced Bipolar and Related Disorder.  DSM-5: Specifier under bipolar and related disorders due to antidepressant or somatic treatment in the context of psychedelic-assisted therapy; relevant for type III bipolar model. |
| Mood switch | Rapid mood elevation (toward mania or hypomania) occurring after psychedelic or serotonergic drug exposure. | Mood change within 24–72h post-use; may include irritability, euphoria, decreased need for sleep. Must persist beyond intoxication to meet diagnostic criteria. | DSM-5 and ICD-11 discuss mood instability post-substance use in context of bipolar spectrum. |
| Psychedelic-Induced dysphoria,  induction of mixed state | Affective state where manic and depressive symptoms co-occur or alternate rapidly, possibly triggered by psychedelics in vulnerable individuals. | Symptoms present concurrently (e.g., agitation + low mood) or fluctuate rapidly. Can emerge acutely within hours to days; may require mood stabilizers. | DSM-5: Specifier for bipolar Disorder ('with mixed features'); relevant to both manic and depressive episodes. Possibility of progressive worsening of cyclicity. |
| **Transition to a bipolar disorder** | | | |
| Transition to bipolar disorder | Development of a full DSM-defined bipolar disorder (usually Bipolar I or II) following an initial psychedelic- or treatment-induced manic/hypomanic episode. | Typically occurs within months to years following a psychedelic-induced episode. May be preceded by multiple mood episodes or conversion per DSM-5 criteria. | DSM-5: Bipolar I/II disorder; transition diagnosed based on recurrence of spontaneous manic/depressive episodes. Supports concept that psychedelics may unmask latent bipolar disorder over time, especially with repeated exposures. |
| Cyclothymia temperament | Underlying biological or familial predisposition to bipolar disorder, which may be unmasked by psychedelic exposure. | Subclinical cyclothymia, family history, or prior antidepressant-induced switch are key indicators. May become apparent after a psychedelic challenge. | Not a formal diagnosis; consistent with cyclothymic temperament or bipolar spectrum vulnerabilities. Supports the staging model of bipolarity; repeated use may reduce threshold for switch. |

**Supplementary Information 1.** Search terms used for our systematic review.

**Embase and Medline**

(bipolar OR 'bipolar disorder'/exp OR 'bipolar disorder' OR 'bipolar affective' OR 'manic depress*' OR cyclothymi* OR 'mixed state*') AND ('psilocybin'/exp OR psilocybin OR psilocyb* OR 'lysergic acid diethylamide'/exp OR 'lysergic acid diethylamide' OR 'lsd'/exp OR lsd OR dmt OR 'n,n-dimethyltryptamine'/exp OR 'n,n-dimethyltryptamine' OR 'ayahuasca'/exp OR ayahuasca OR 'mescaline'/exp OR mescaline OR 'peyote'/exp OR peyote OR 'ibogaine'/exp OR ibogaine OR psychedelic* OR hallucinogen* OR 'mdma'/exp OR mdma) AND ('mania'/exp OR mania OR manic OR 'hypomania'/exp OR hypomania OR hypomanic OR 'manic episode'/exp OR 'manic episode' OR 'hypomanic episode'/exp OR 'hypomanic episode' OR 'treatment emergent mania' OR 'affective switch' OR 'treatment-emergent affective switch' OR 'induced mania' OR 'precipitated mania' OR 'mood switch' OR 'treatment emergent')

**Pubmed**

(("bipolar"[MeSH] OR "bipolar disorder"[tiab] OR "bipolar affective"[tiab] OR "manic depress*"[tiab] OR cyclothymi*[tiab] OR "mixed state*"[tiab]) AND ("psilocybin"[MeSH] OR psilocybin[tiab] OR psilocyb*[tiab] OR "lysergic acid diethylamide"[MeSH] OR "lysergic acid diethylamide"[tiab] OR lsd[tiab] OR dmt[tiab] OR "n,n-dimethyltryptamine"[tiab] OR "ayahuasca"[tiab] OR "mescaline"[MeSH] OR mescaline[tiab] OR peyote[tiab] OR ibogaine[tiab] OR psychedelic*[tiab] OR hallucinogen*[tiab] OR "mdma"[MeSH] OR mdma[tiab]) AND ("mania"[MeSH] OR mania[tiab] OR manic[tiab] OR "hypomania"[MeSH] OR hypomania[tiab] OR hypomanic[tiab] OR "manic episode"[MeSH] OR "manic episode"[tiab] OR "hypomanic episode"[MeSH] OR "hypomanic episode"[tiab] OR "treatment emergent mania"[tiab] OR "affective switch"[tiab] OR "treatment-emergent affective switch"[tiab] OR "induced mania"[tiab] OR "precipitated mania"[tiab] OR "mood switch"[tiab] OR "treatment emergent"[tiab]))

**Supplementary Figure 1.** Prisma 2020 Flow diagram.

**Identification of studies via databases and registers**

Records removed *before screening*:

Duplicate records removed (n = 112)

Records marked as ineligible by automation tools (n = 0)

Records removed for other reasons (n = 0)

Records identified from*:

Databases (n = 684)

Registers (n = 60)

**Identification**

Records excluded** (n = 575:

Case reports (n = 51)

Systematic reviews (n = 36)

Not relevant (n = 488)

Records screened

(n = 632)

Reports sought for retrieval

(n = 57)

Reports not retrieved

(n = 0)

**Screening**

Reports excluded:

Excluded due to study design (case reports/series): (n = 11)

Excluded due to absence of relevant bipolar/manic outcomes: (n = 16)

Excluded due to lack of original or extractable data: (n = 7)

Reports assessed for eligibility

(n = 57)

Studies included in review

(n = 23) *19 published studies and 4 ongoing/terminated controlled trials*

Reports included in the meta-analysis

(n = 4)

**Included**

*Consider, if feasible to do so, reporting the number of records identified from each database or register searched (rather than the total number across all databases/registers).

**If automation tools were used, indicate how many records were excluded by a human and how many were excluded by automation tools.

Source: Page MJ, et al. BMJ 2021;372:n71. doi: 10.1136/bmj.n71.

**Supplementary Table 2.** Excluded studies from our systematic review, when assessed for eligibility.

| **Study / DOI** | **Study type** | **Population / Exposure** | **Reason for exclusion** |
| --- | --- | --- | --- |
| **Barbic et al., 2020** 10.1016/j.ajem.2020.04.090 | Retrospective observational (ED presentations) | Acute intoxication cases | Focused on emergency presentations; no data on hypomania/mania or bipolar outcomes |
| **Bickel et al., 2005** 10.1097/00063110-200512000-00011 | Observational / commentary | Substance use | No data on psychedelic- or MDMA-induced manic or bipolar outcomes |
| **Br. Med. J., 1979** 2(6193):797 | Commentary / brief report | Hallucinogen use | No systematic data on mood switches or bipolar diagnosis |
| **Brown et al., 2017** 10.1080/02791072.2017.1344898 | Case report | Individual case | Case report excluded per eligibility criteria |
| **Cohen, 1966** 10.1016/S0033-3182(66)72149-5 | Narrative review | Hallucinogens | Historical review; no original data meeting inclusion criteria |
| **Davies, 1979** | Narrative review | Hallucinogenic compounds | Descriptive review; no extractable manic/bipolar outcomes |
| **Dewhurst, 1980** 10.1192/bjp.137.3.303a | Commentary | Hallucinogens | No original data; anecdotal observations only |
| **Dos Santos et al., 2017** | Narrative review | Ayahuasca | Review of effects; no primary data on mania/bipolar outcomes |
| **Szmulewicz et al., 2015** 10.1186/s40345-014-0020-y | Case report | Individual case | Case report excluded per eligibility criteria |
| **Neubiorev., 2019** 10.1016/j.neubiorev.2019.12.001 | Narrative review | Psychedelics | No original data on substance-induced mania or bipolar disorder |
| **Hendin & Penn, 2021** 10.1111/bdi.13095 | Case report | Individual case | Case report excluded per eligibility criteria |
| **Hyde et al., 1978** 10.1192/bjp.132.6.602 | Case report / brief series | Individual cases | Case-based evidence only |
| **Lake et al., 1981** 10.1176/ajp.138.11.1508 | Case report | Individual case | Case report excluded per eligibility criteria |
| **Paterson et al., 2015** 10.1097/WNF.0000000000000078 | Case report | Individual case | Case report excluded per eligibility criteria |
| **Perera et al., 1995** 10.1080/00048679509075930 | Case report | Individual case | Case report excluded per eligibility criteria |
| **Prajapati et al., 2016** 10.5455/bcp.20151217105437 | Case report | Individual case | Case report excluded per eligibility criteria |
| **Reich & Hepps, 1972** 10.1001/jama.1972.03190330039008 | Case report | Individual case | Case report excluded per eligibility criteria |
| **Sami et al., 2015** 10.3109/02699052.2015.1046491 | Cross-sectional observational | General population | Did not assess manic/hypomanic or bipolar outcomes following psychedelics |
| **10.1080/17523281.2010.509845** | Cross-sectional survey | Students; MDMA | No significant association with hypomanic symptoms; not bipolar-focused |
| **10.3109/14659890903060167** | Case report | Individual case | Case report excluded per eligibility criteria |
| **10.1192/bjp.175.5.467** | Cross-sectionnal study | Drugs associated to a bipolar disorder. | No clear data on substance-induced mania |
| **10.1186/s40345-014-0020-y** | Case report | Individual case | Case report excluded per eligibility criteria |
| **Estroff et al., 1986** | Cohort study | Cohort | Case report excluded per eligibility criteria |
| **10.1176/ajp.155.5.646** | Cohort study | Cohort | Case report excluded per eligibility criteria |
| **10.1080/02791072.2017.1344898** | Case report | Individual case | No data on substance-induced mania |
| **10.1192/bjp.187.6.510** | Narrative review | Not specified | No original data on psychedelic-induced mania |
| **10.1001/jamapsychiatry.2023.1256** | Commentary / review | Not specified | No original data on manic or bipolar outcomes |
| **10.1111/add.15881** | Narrative review | Substance use | No data on substance-induced mood disorders |
| **10.4088/jcp.v60n1103** | Review article | Mood disorders | No data on substance-induced mania |
| **10.1002/psb.2148** | Not relevant | Other drugs | No data on substance-induced mania |
| **10.1007/s00406-024-01925-6** | Not relevant | Other drugs | No data on substance-induced mania |
| **10.1007/BF03259908** | Narrative review of case reports | Mood disorders | Case series |
| **10.1055/a-2529-7029** | Narrative review | Substance use | No data on substance-induced mood disorders |
| **10.1177/02698811241278873** | Review article | Mood disorders | No data on substance-induced mania |
| [**10.1016/j.jad.2026.121220**](https://doi.org/10.1016/j.jad.2026.121220) | Qualitative anylsis of online Forum | Mood symptoms | Qualitative anlysis |
| **10.1177/02698811241265762** | Narrative review | Substance use | No data on substance-induced mood disorders |
|  |  |  |  |

**Supplementary Table 3.** Risk of bias assessment using ROBINS-I, ROB2 or NIH.

| **ROBINS-I (Risk Of Bias In Non-randomized Studies - of Interventions)** | | | | | | | | | | |
| --- | --- | --- | --- | --- | --- | --- | --- | --- | --- | --- |
| **Study** | **Design / Sample** | **Confounding** | **Selection into study** | **Classification of exposure** | **Departure from intended exposure** | **Measurement of outcomes** | **Missing data** | **Selection of reported results** | **Overall risk of bias** |  |
| **1.Aaronson S. T. et al., 2023** | Non-randomised open-label trial; n = 15 adults (18–65) with treatment-resistant bipolar II depression | Serious – No control group; strong potential confounding from expectancy effects, psychotherapy component, illness course, regression to the mean | Moderate – Highly selected sample (volunteers, strict inclusion criteria, single centre) | Low – Exposure clearly defined (single 25 mg dose of synthetic psilocybin + psychotherapy) | Low – No evidence of deviation from protocol; supervised administration | Moderate – Outcomes based on validated scales (MADRS, YMRS, CSSRS) but assessor and participants unblinded | Low – Minimal attrition; outcome data available for most participants | Moderate – Selective emphasis on short-term efficacy; no comparator outcomes | **Moderate to Serious risk of bias** |  |
| **2.DellaCrosse et al., 2022** | Retrospective observational study / self-reported psychedelic use among individuals with bipolar disorder (online recruitment) | Serious – No control group; strong confounding by indication, expectancy effects, illness severity, concurrent treatments, substance use history, and natural mood cycling | Serious – Convenience, self-selected online sample; high risk of selection on outcome and exposure; limited representativeness | Serious – Exposure retrospectively self-reported; substance identity, dose, purity, timing, and context variably defined; misclassification likely | Moderate – No assigned exposure; variability in setting, dosing, and co-interventions; deviations inherent to naturalistic use | Serious – Outcomes self-reported without structured clinical interviews; manic/hypomanic symptoms not systematically adjudicated; recall bias | Moderate – Incomplete responses and variable follow-up; handling of missing data limited. | Moderate – Exploratory analyses; selective emphasis on salient or positive findings possible; no preregistered analysis plan | **Serious risk of bias** |  |
| **3.Marrocu et al., 2024** | Prospective observational naturalistic study; pooled online cohorts; n = 807 adults reporting self-initiated psychedelic use | Serious – No randomization or control group; strong residual confounding from baseline mental health status, expectancy effects, concurrent psychotherapy | Serious – Self-selected participants recruited online; individuals intending to use psychedelics; exclusion of those with severe adverse experiences may bias sample | Moderate – Exposure self-reported; substance type, dose, and context variably specified; no biological verification | Low – Naturalistic design; no protocol-defined intended exposure; no evidence of systematic deviation once exposure occurred | Moderate to Serious – Outcomes based on self-reported mental well-being measures; no clinician-rated assessments; no structured evaluation of manic, hypomanic, or psychotic episodes | Moderate – Attrition between baseline and 4-week follow-up; missingness likely non-random | Moderate – Pre-specified primary outcome reported; limited subgroup reporting (e.g., bipolar disorder not stratified); emphasis on overall well-being change | **Serious** |  |
| **4.Meyer T.D. et al., 2025** | Retrospective observational naturalistic study; adults with diagnosed bipolar disorder reporting recent recreational psychedelic use; within-subject pre–post comparison | Serious – No control group; strong residual confounding (expectancy effects, natural illness fluctuation, concurrent treatments, concurrent substance use including cannabis) | Serious – Self-selected participants willing to disclose psychedelic use | Moderate – Exposure based on retrospective self-report; substance, dose, purity, and context variably specified; timing anchored but not biologically verified | Low – Naturalistic exposure; no intended protocol from which to deviate; exposure reflects real-world use | Moderate – Outcomes based on self-reported symptom days via Timeline Follow-back; no blinded assessment; limited objective verification of manic or psychotic symptoms | Moderate – Retrospective recall over several months; potential incomplete or imprecise reporting of symptom days and substance use | Moderate – Primary emphasis on depressive symptoms and symptom-free days; limited reporting of rare but clinically salient adverse outcomes | **Serious risk of bias** |  |
| **5.Honk et al., 2024** | Longitudinal observational; population-based adult samples in US and UK (n≈9,732) | Serious — confounding likely, not fully controlled | Moderate–Serious — participation and follow-up could differ by health status; not randomized | Moderate — self-reported psychedelic use; dose/type/timing not verified | Not applicable — exposure is naturalistic and observational | Moderate — psychotic symptoms self-reported; clinical confirmation limited | Moderate — some loss to follow-up possible | Low–Moderate — main outcomes reported; subgroup analyses exploratory | **Serious** |  |
| **6.Meshkat et al., 2025** | Non-randomized open-label case series; adults with treatment-resistant BD-II | Serious – No control group; confounding by psychotherapy, expectancy effects, illness course | Moderate – Selected clinical population; inclusion based on treatment resistance and consent | Low – Exposure clearly defined (1–2 sessions, 25 mg psilocybin + psychotherapy) | Low – Intervention delivered as intended; no protocol deviations reported | Moderate – Validated scales (MADRS, YMRS) but unblinded assessments | Low – Follow-up data reported up to 6 months | Moderate – Focus on efficacy and safety outcomes; limited reporting of negative or null effects | **Moderate to Serious risk of bias** |  |
| **7.Meyer et al., 2025** | Recreational use in individuals with bipolar disorder (naturalistic TLFB) | Moderate–Serious – potential confounding by mood state, medication, and baseline symptoms; not controlled | Serious – self-selected convenience sample of people with BD | Moderate – self-reported recent use; recall calendar (TLFB) method improves timing but remains subjective | N/A – not intervention | Moderate – mood symptoms measured pre/post use but no clinical diagnosis required | Moderate – missing calendar data possible over 3 months | Moderate – outcomes largely self-reported; no preregistered hypotheses noted | **Serious** |  |
| **8.Rognli et al., 2023** | Nationwide registry-based cohort; Norway; N = 3,187 with substance-induced psychosis | Moderate – Residual confounding (polysubstance use, treatment, psychosocial factors) | Low – National patient registry ensures broad inclusion | Low – Exposure classified via standardized registry diagnoses | Low – No deviations expected | Low – Long-term outcomes captured via national registers | Low – Registry data ensures near-complete follow-up | Low – Outcomes reported transparently with confidence intervals | **Moderate risk of bias** |  |
| **9.Rosenblat J.D. et al., 2024** | Observational study using secondary data / survey-based or registry-based assessment of psychedelic exposure and mood outcomes | Serious – Strong residual confounding ; no randomization or adequate adjustment for illness course | Moderate–Serious – Sample selection dependent on data source and participation; potential selection on exposure and outcome; limited representativeness | Moderate – Exposure based on self-report or secondary records; dose, timing, frequency, and context incompletely characterized | Moderate – No assigned exposure; naturalistic use with heterogeneous dosing and co-interventions; deviations inherent to design | Moderate–Serious – Outcomes based on symptom scales or diagnostic proxies; limited structured adjudication of manic/hypomanic episodes; potential misclassification | Low–Moderate – Missingness present but not clearly differential; handling described but limited | Moderate – Selective emphasis on associations of interest; no preregistered causal analysis plan | **Serious risk of bias** |  |
| **10.Simonsson et al., 2025 (Psychological Medicine)** | Longitudinal observational, US adults (baseline N≈21,990; follow-up 12,345) | Serious – residual confounding possible despite adjustments; exposure non-random | Serious – self-selected follow-up; retention issues (56%) | Moderate – self-reported psychedelic use; context (illegal/legal) | Low–Moderate – exposure during period measured; not assigned by researchers | Moderate – symptom scales self-reported without clinical interview | Moderate – 44% attrition at follow-up | Moderate – analytic flexibility with subgroup effects | **Serious** |  |
| **11.Starzer et al., 2018** | Nationwide registry-based cohort; Denmark; N = 6,788 individuals with substance-induced psychosis | Moderate – Residual confounding likely (severity of substance use, genetic vulnerability, treatment exposure) | Low – Population-based national registers minimize selection bias | Low – Exposure (substance-induced psychosis) defined using standardized diagnostic codes | Low – Observational design; no intervention deviations | Low – Outcomes (bipolar disorder, schizophrenia) ascertained via validated national registers | Low – Minimal loss to follow-up due to registry linkage | Low – Outcomes predefined and systematically reported | **Moderate risk of bias** |  |
| **12.Wirowski et al., 2025** | Registry-based cohort; N = 468 adults (18–60) with MDD | Serious – Limited control for confounding; substance use history self-reported / incompletely captured | Moderate – Clinical cohort rather than population-wide; possible selection bias | Moderate – Lifetime hallucinogen use likely underreported; misclassification possible | Low – No deviation issues in observational design | Moderate – Bipolar conversion registry-based, but exposure measurement less robust | Moderate – Small sample; incomplete capture of lifetime substance exposure possible | Moderate – Limited power; selective emphasis on subgroup findings | **Moderate to Serious risk of bias** |  |
| **13. Vale et al. 2026**  **(part of Meyer et al. 2025 study)** | Observational naturalistic study; adult participants with mood-disorder vulnerability exposed to psychedelics in non-randomized settings | Serious – Non-randomized exposure; strong potential confounding by indication, expectancy effects, illness severity, concomitant treatments, and contextual factors | Serious – Self-selected participants; recruitment likely biased toward individuals with prior psychedelic experience or favorable attitudes; limited representativeness | Moderate – Exposure based on self-reported psychedelic use; substance type, dose, purity, and timing may be imprecisely classified | Low – Naturalistic design; no protocol-defined exposure from which to deviate | Moderate – Outcomes based on self-reported manic, hypomanic, or psychotic symptoms; validated scales used, but no blinded assessment and no clinician-rated diagnostic confirmation | Moderate – Attrition and incomplete follow-up typical of online or community cohorts; missingness may be related to symptom severity | Moderate – Multiple symptom outcomes assessed; risk of selective emphasis on statistically significant findings cannot be excluded | **Serious risk of bias** |  |
| **14. Myran et al. 2025** | Population-based registry cohort study; province-wide administrative health data; individuals receiving hospital-based care for hallucinogen-related conditions, with longitudinal follow-up for incident mania and bipolar disorder diagnoses | Moderate – Adjusted for multiple sociodemographic and clinical covariates; residual confounding likely due to polysubstance use, illness severity, prodromal psychiatric symptoms, and indication for hospital presentation | Low – Population-based inclusion using comprehensive administrative registers; minimal risk of selection bias at cohort entry | Moderate – Exposure defined by hospital-based care for hallucinogen-related diagnoses; accurately captured but represents a severe and selected subset of users rather than hallucinogen use per se | Low – Observational registry design; no intended exposure protocol and no deviation concerns | Low – Outcomes based on clinically recorded diagnoses of mania and bipolar disorder in administrative health records; high specificity and temporal ascertainment | Low – Minimal missing outcome data due to use of linked population-level administrative databases | Low to Moderate – Prespecified outcomes and analyses reported; limited risk of selective reporting, though subgroup and time-window analyses may emphasize early risk periods | **Moderate risk of bias** |  |
| **NIH Quality Assessment Tool – Cross-Sectional Studies)** | | | | | | | | | | |
| **Study** | **Study design / sample** | | **Appropriate appraisal tool** | | **Key bias domains assessed** | | **Overall methodological appraisal** | | | |
| **1. Morton et al., 2023** | Cross-sectional international web-based survey (self-reported bipolar disorder; n≈541) | | NIH Quality Assessment Tool – Cross-Sectional Studies | | Sampling and selection bias (self-selected convenience sample), exposure measurement (self-reported psilocybin use; dose/timing uncertainty), outcome measurement (self-reported symptom change without diagnostic confirmation), recall bias, missing data | | Serious risk of bias (non-probability sampling; no temporality; exploratory, descriptive outcomes; not suitable for causal inference) | | | |
| **2.Simonsson et al., 2025 (NSDUH)** | Cross-sectional population survey (US adults) | | NIH Quality Assessment Tool – Cross-Sectional Studies | | Sampling frame, measurement validity, non-response bias | | Serious risk of bias for causality (no temporality; self-report exposure/outcomes) | | | |
| **3.Simonsson et al., 2024 (JAMA Psychiatry)** | Cross-sectional adolescent twin sample | | NIH Quality Assessment Tool – Cross-Sectional Studies | | Selection bias, shared confounding, measurement error | | Serious risk of bias (cross-sectional; limited psychiatric control) | | | |

| **Risk of Bias (RoB 2) – Domain-Level Assessment** | | | | | | |
| --- | --- | --- | --- | --- | --- | --- |
| **Study** | **D1: Bias arising from the randomization process** | **D2: Bias due to deviations from intended interventions** | **D3: Bias due to missing outcome data** | **D4: Bias in measurement of the outcome** | **D5: Bias in selection of the reported result** | **Overall RoB 2 judgment** |
| **Bogenschutz et al., 2022** | Low risk – adequate random sequence generation and allocation concealment | Low risk – double-blind design with active placebo; no evidence of differential deviations | Low risk – low attrition; missing data balanced and appropriately handled | Low risk – validated outcome measures; blinded assessors | Low risk – outcomes prespecified and reported as planned | **Low risk of bias** |
| **Goodwin et al., 2022** | Low risk – centralized randomization with adequate concealment | Some concerns – probable functional unblinding due to marked psychoactive effects | Some concerns – non-negligible attrition at follow-up, though balanced | Low risk – standardized clinician-rated scales with blinded raters | Low risk – outcomes aligned with prespecified analysis plan | **Some concerns** |

**Notes :**

*ROBINS-E judgments reflect risk of bias, not study quality or importance. The overall judgment corresponds to the highest level of bias across critical domains, particularly confounding and temporality.

*Cross-sectional studies were assessed using the NIH Quality Assessment Tool for Observational Cohort and Cross-Sectional Studies. Ratings focused on selection bias, measurement validity, and confounding. Items related to temporality and follow-up were considered not applicable due to study design. Overall study quality was judged qualitatively (good, fair, or poor) based on critical risk-of-bias domains rather than a numeric score

*RoB 2 Judgments

a. Randomization (D1): Low risk when sequence generation and allocation concealment were adequate.

b. Deviations (D2): Some concerns reflect likely functional unblinding due to psychoactive effects, despite formal blinding and active placebo.

c. Missing data (D3): Low risk with minimal/balanced attrition; some concerns when follow-up loss was non-negligible.

d. Outcome measurement (D4): Low risk with validated measures and blinded assessors.

e. Selective reporting (D5): Low risk when outcomes and analyses were prespecified and consistently reported.

f. Overall RoB 2: Determined by the highest risk level across domains.

g. Safety outcomes: Trials not powered to detect rare manic/hypomanic switches.

**Supplementary Figure 2.** Funnel plot of the prevalence meta-analysis with all included studies.

**Supplementary Figure 3.** Forest plot of the proportion of transition to bipolar disorder following hallucinogen-induced psychosis or mood disorder (random-effects logit model) with exclusion of Myran et al. 2025 study.
